# Insight into contact force local impedance technology for predicting effective pulmonary vein isolation

**DOI:** 10.3389/fcvm.2023.1169037

**Published:** 2023-07-05

**Authors:** Antoine Lepillier, Ruggero Maggio, Valerio De Sanctis, Maurizio Malacrida, Giuseppe Stabile, Cyril Zakine, Laure Champ-Rigot, Matteo Anselmino, Luca Segreti, Gabriele Dell’Era, Fabien Garnier, Giuseppe Mascia, Claudio Pandozi, Antonio Dello Russo, Marco Scaglione, Giuseppe Cosaro, Anna Ferraro, Olivier Paziaud, Giampiero Maglia, Francesco Solimene

**Affiliations:** ^1^ Centre Cardiologique du Nord, Saint-Denis, France; ^2^ Infermi Hospital, Rivoli, Italy; ^3^ IRCCS Ospedale Galeazzi—Sant’Ambrogio, Milan, Italy; ^4^ Boston Scientific, Milan, Italy; ^5^ Mediterranea Cardiocentro, Napoli, Italy; ^6^ Clinique NCT, St Cyr sur Loire, France; ^7^ Normandie Univ, UNICAEN, CHU de Caen Normandie, Caen, France; ^8^ Division of Cardiology, “Città della Salute e della Scienza di Torino” Hospital, Department of Medical Sciences, University of Turin, Turin, Italy; ^9^ Second Division of Cardiology, Cardiac-Thoracic-Vascular Department, New Santa Chiara Hospital, Azienda Ospedaliero Universitaria Pisana, Pisa, Italy; ^10^ Azienda Ospedaliera Universitaria “Maggiore della Carità”, Novara, Italy; ^11^ Centre Hospitalier de Dijon Bourgogne, Dijon, France; ^12^ IRCCS San Martino Polyclinic Hospital, Genoa, Italy; ^13^ San Filippo Neri Hospital, Roma, Italy; ^14^ Cardiology and Arrhythmology Clinic, Marche Polytechnic University, Ancona, Italy; ^15^ Cardinal Massaia Hospital, Asti, Italy; ^16^ Azienda Ospedaliera Pugliese Ciaccio, Catanzaro, Italy; ^17^ Department of Cardiac Electrophysiology and Arrhythmology, Clinica Montevergine, Mercogliano, Italy

**Keywords:** atrial fibrillation, catheter ablation, local impedance, contact force, lesion formation, pulmonary vein isolation

## Abstract

**Background:**

Highly localized impedance (LI) measurements during atrial fibrillation (AF) ablation have the potential to act as a reliable predictor of the durability of the lesions created.

**Objective:**

We aimed to collect data on the procedural parameters affecting LI-guided ablation in a large multicenter registry.

**Methods:**

A total of 212 consecutive patients enrolled in the CHARISMA registry and undergoing their first pulmonary vein (PV) isolation for paroxysmal and persistent AF were included.

**Results:**

In all, 13,891 radiofrequency (RF) applications of ≥3 s duration were assessed. The first-pass PV isolation rate was 93.3%. A total of 80 PV gaps were detected. At successful ablation spots, baseline LI and absolute LI drop were larger than at PV gap spots (161.4 ± 19 Ω vs. 153.0 ± 13 Ω, *p* < 0.0001 for baseline LI; 22.1 ± 9 Ω vs. 14.4 ± 5 Ω, *p* < 0.0001 for LI drop). On the basis of Receiver operating characteristic curve analysis, the ideal LI drop, which predicted successful ablation, was >21 Ω at anterior sites and >18 Ω at posterior sites. There was a non-linear association between the magnitude of LI drop and contact-force (CF) (*r* = 0.14, 95% CI: 0.13–0.16, *p* < 0.0001) whereas both CF and LI drop were inversely related with delivery time (DT) (−0.22, −0.23 to −0.20, *p* < 0.0001 for CF; −0.27, −0.29 to −0.26, *p* < 0.0001 for LI drop).

**Conclusion:**

An LI drop >21 Ω at anterior sites and >18 Ω at posterior sites predicts successful ablation. A higher CF was associated with an increased likelihood of ideal LI drop. The combination of good CF and adequate LI drop allows a significant reduction in RF DT.

**Clinical trial registration:**

http://clinicaltrials.gov/, identifier: NCT03793998.

## Introduction

Pulmonary vein (PV) isolation is the cornerstone of atrial fibrillation ablation (AF). The likelihood of obtaining permanent PV isolation is related to the quality of ablation energy delivery and lesion formation. With radiofrequency (RF) energy, it is known that catheter stability, contact force (CF), power output, temperature and duration affect the size and transmurality of the lesion created (1). Generator impedance (GI) drop, defined as the difference between the minimum impedance value during RF application and the baseline impedance, encompasses the effects of several of these parameters, and has, indeed, been used as a surrogate of ablation efficacy (2–5). Recently, new ablation catheters have enabled the possibility to measure highly localized impedance (LI) during AF ablation: this innovative real-time measurement has the potential to act as viable indicator of tissue characteristics and a reliable predictor of the durability of the lesions created (6–9).

The aim of the present study was to collect data on all contact force local impedance technology parameters in a large, international, multicenter registry on AF ablation, in order to assess their relationships with effective PV isolation.

## Methods

### Patient population and study design

CHARISMA is a prospective, multicenter cohort study designed to collect data on clinical practice concerning the ablation approach to various arrhythmias. The study complies with the Declaration of Helsinki; the locally appointed ethics committee approved the research protocol, and informed consent was obtained from all patients. From June 2021 to June 2022, 212 consecutive patients referred for paroxysmal and persistent AF ablation and undergoing their first high-resolution mapping and ablation procedure with a novel CF- and LI-featured catheter in 16 European centers were included. Of these, twenty cases (out of 45) with complete information were analyzed from our previous pilot study (9). All patients were followed up at the same hospital, from the time of first ablation to the last follow-up visit.

### Ablation procedure

The ablation procedure was adapted on our pilot experience in order to standardize each operator's approach (9). Briefly, all procedures were performed under conscious sedation or general anesthesia. Vitamin K antagonist treatment was not interrupted, while non-vitamin K anticoagulants were withheld on the morning of the procedure. A decapolar catheter was used to cannulate the coronary sinus. After single or double transseptal punctures under fluoroscopic guidance, intravenous unfractionated heparin boluses were administered, in order to maintain an activated clotting time of >300 s. The basket mapping catheter (Orion™, Boston Scientific, Marlborough, MA, USA) and the ablation catheter (Stablepoint™ catheter, Boston Scientific, Marlborough, MA, USA) were then inserted. A standard, steerable (i.e., Agilis®, Abbott) or non-steerable sheath was used. The Orion™ catheter was used in combination with the Rhythmia™ HDx mapping system (Rhythmia™, Boston Scientific, Marlborough, MA, USA) to create a 3-dimensional electro-anatomical voltage and activation map of the left atrium. Mapping and ablation were primarily carried out in sinus rhythm; in patients in AF, electrical cardioversion was attempted in order to restore sinus rhythm, at the beginning of the procedure and before re-mapping. Point-by-point RF delivery was performed in such a way as to create contiguous ablation spots encircling the PVs. CF settings were at the individual operator's discretion, within the range of 5–40 g. Ablation was guided by the magnitude and time course of the impedance drop during RF delivery. RF applications were targeted to a minimum LI drop of 15 Ω within 15 s and were stopped when a maximum cutoff LI drop of ≥40 Ω was observed. On the basis of previous experimental data, the general indication was to reach an LI drop of 20–30 Ω (10). Radiofrequency energy was applied in the power-controlled mode (45–50 W) with a temperature limit of 43°C. The irrigation rate, with normal saline solution (NaCl 0.9%), was 30 ml/min during applications and 2 ml/min during mapping. The recommended maximum distance between adjacent ablation spots (center-to-center) was ≤6 mm. The ablation points were marked automatically with 6-mm diameter and numbered by means of AutoTags™. The baseline LI, LI drop and percentage LI drop during RF were recorded. The endpoint of ablation was PV isolation, as assessed on the basis of entry and exit block by means of the 64-pole Orion™ catheter placed sequentially in each PV. In the absence of first-pass PV isolation (i.e., no isolation upon complete encirclement of ipsilateral veins), PV isolation was accomplished by means of additional RF applications at the investigator's discretion.

### Local impedance

A 3-electrode method with separate circuits for field creation and measurement was used to measure LI. As described elsewhere, non-stimulatory alternating current was delivered between the tip electrode and the proximal ring; voltage was passively measured between the tip electrode and the distal ring (11). As the study catheter does not have mini-electrodes, the resulting voltage was measured from the catheter tip. Impedance was calculated by dividing the voltage by the stimulatory current. In a pilot study, we defined a standardized approach to record and analyze local impedance information (9). Briefly, to measure the baseline reference impedance of the blood pool, once the reference map had been completed, the ablation catheter was positioned in the blood pool for 10 s, and the value was calculated when no EGM recordings from the ablation catheter were present. Baseline tissue impedance and impedance drop for each ablation lesion were measured. To analyze the impedance information, the isolation line around each pair of PVs was divided into seven distinct sections, in accordance with previous literature (12). RF current applications were then retrospectively analyzed. All numbered AutoTag^TM^ points were exported from the system for off-line analysis.

### Contact force

The ablation catheter used in the current study, in addition to measuring LI from a local electric field generated at the tip of the catheter, has the ability to monitor real-time CF. The force applied to the tip electrodes is transferred to inductive sensors via a spring. The signal change measured by the inductive sensors is then converted to a 3-dimensional force vector by means of known spring dynamics. The target CF was 5–40 g, at the operator's discretion. The following data on each first-pass ablation point were collected: power, minimum CF, maximum CF and mean CF. In addition, the CF range during the applications was calculated by subtracting the minimum CF from the maximum CF at the ablation point.

### Statistical analysis

Continuous data are expressed as mean ± standard deviation or median values with interquartile range, as appropriate, for all the variables. Continuous variables were compared by means of Student's *t*-test, analysis of variance, or non-parametric test (median test or Mann–Whitney *U*-test), as appropriate. Categorical data were compared by means of the v2 test (Pearson, Yates or Fisher's exact test, as appropriate). To determine relationships among CF, RF DT and LI drop a linear regression analysis was applied. Logistic regression was performed to determine the parameters associated with successful applications (i.e., first-pass isolation). A probability value of *p* less than 0.05 was considered statistically significant. To determine the ideal LI drop, able to distinguish acutely successful ablation applications from ineffective ones, a receiver-operator characteristic (ROC) curve was constructed. Youden's index was used to determine the optimal LI drop cutoff value. All statistical analyses were performed by means of STATISTICA software, version 7.1 (StatSoft, Inc., Tulsa, OK).

## Results

### Study population and procedural parameters

Patient characteristics and procedural data are summarized in Table 1. Briefly, 130 (61.3%) patients had paroxysmal and 82 (38.7%) persistent AF. Most patients were men (*n* = 146, 68.9%) and the mean age was 61 ± 10 years. The mean procedure duration and fluoroscopy times were 115 ± 41 and 10 ± 7 min, respectively. A total of 15,599 RF applications were delivered, with a mean number of 68 ± 23 ablation spots, during a mean RF duration time of 9.2 ± 4 s, without any steam popping. No case of tamponade, stroke or esophageal fistula occurred.

**Table 1:** Clinical characteristics of the study population.

Parameter	*n* = 212
Age, years	61 ± 10
Male Gender, *n* (%)	146 (68.9)
Indication for ablation • Paroxysmal AF, *n* (%) • Persistent AF, *n* (%)	• 130 (61.3) • 82 (38.7)
History of atrial flutter/atrial tachycardia, *n* (%)	44 (20.8)
LVEF, %	56 ± 10
Cardiomyopathy, *n* (%)	55 (25.9)
Hypertension, *n* (%)	105 (49.5)
Coronary artery disease, *n* (%)	28 (13.2)
History of heart failure, *n* (%)	36 (17.0)
COPD, *n* (%)	9 (4.2)
CKD, *n* (%)	12 (5.7)
ACE-ARB, *n* (%)	79 (37.3)
Beta-blockers, *n* (%)	124 (59.0)
Antiarrhythmics, *n* (%)	144 (67.9)
Procedure duration, min	115 ± 41
Fluoroscopy time, min	10.3 ± 7
RF applications, *n*	68 ± 23
RF duration time, s	9.2 ± 4
Mean Power, W	48 ± 2
Complications during the procedure, *n* (%)	0 (0)
Minor complications • Vascular complications • Pericarditis	7 (3.3) • 4 • 3

### Local tissue impedance values

**Figure 1:**
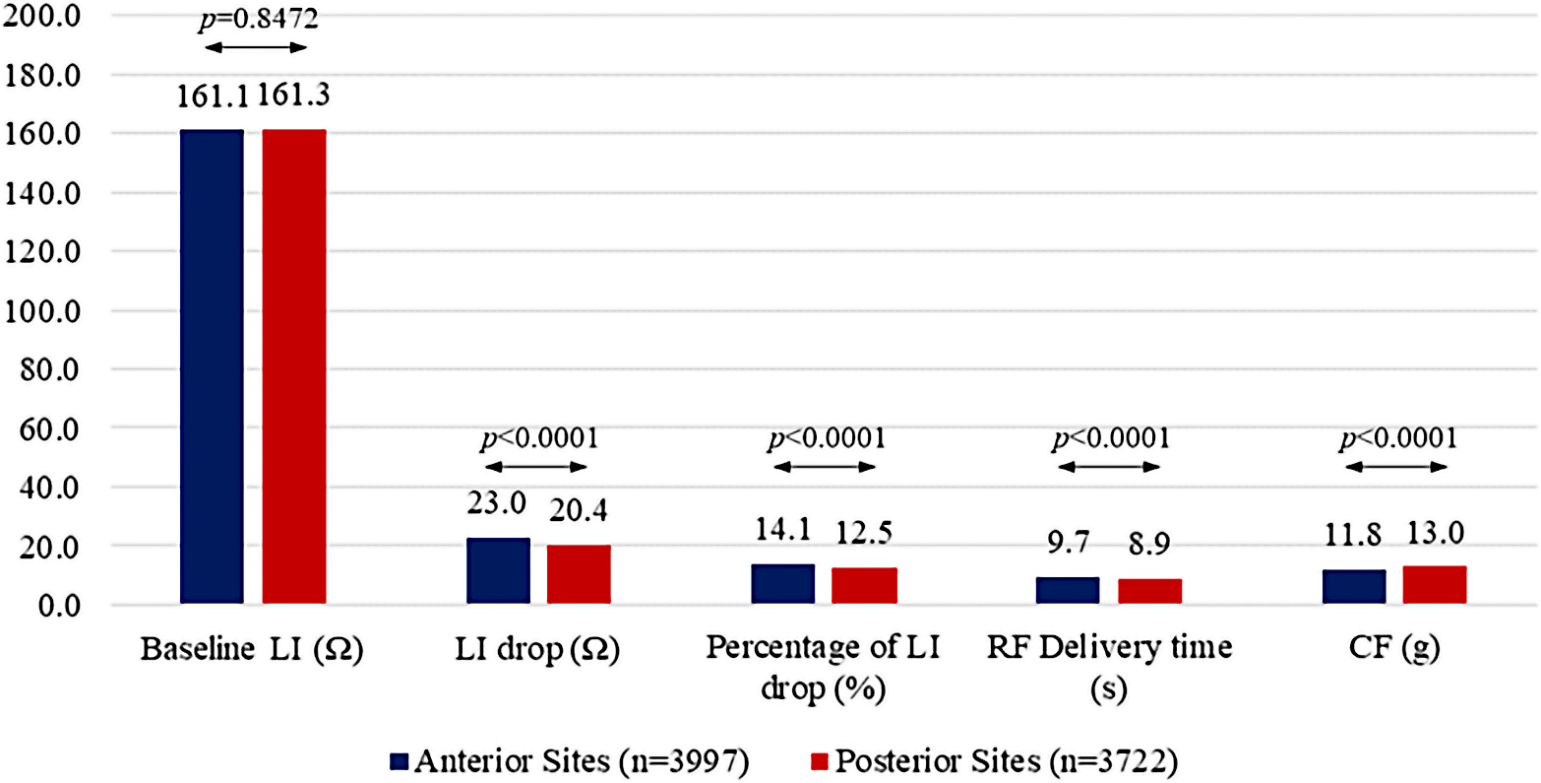
Procedural parameters according to ablation site (anterior vs. posterior).

A total of 13,891 (89.5%) high-quality RF applications of ≥3 s duration around PVs were assessed. LI prior to ablation was 161.2 ± 19 Ω (139.2 ± 15 Ω after ablation, *p* < 0.0001), the absolute LI drop was 21.9 ± 9 Ω (LI drop rate of 3.2 ± 2 Ω/s) and the percentage LI drop was 13.5 ± 5%. The majority (*n* = 10,266, 74%) of RF applications resulted in an LI drop greater than 15 Ω within 15 s, and in about half of the applications (*n* = 7,742, 56%) it was achieved within 10 s. Both the magnitude of the impedance drop and the LI drop rate were predicted by baseline LI [correlation coefficient r 0.56, confidence interval (CI): 0.55–0.57 for absolute LI drop, *p* < 0.0001; *r* = 0.55, 95% CI: 0.53–0.56, *p *< 0.0001 for LI drop rate, *p* < 0.0001]. Of the 13,891 RF applications, 7,421 (53.4%) were to the right PVs and 6,470 (46.6%) to the left PVs. Key parameters were also stratified by left atrial anatomical region. Baseline impedance was homogenous between anterior and posterior sites (161.1 ± 19 Ω at anterior vs. 161.3 ± 18 Ω at posterior sites, *p *= 0.8472) whereas other parameters differed: LI drops were greater (23.0 ± 8 Ω vs. 20.4 ± 9 Ω, *p *< 0.0001), percentages of LI drop were higher (14.1 ± 4% vs. 12.5 ± 4%, *p *< 0.0001), RF delivery times were longer (9.7 ± 4 s vs. 8.9 ± 4 s, *p *< 0.0001) and CF values were lower (11.8 ± 7 g vs. 13.0 ± 7 g, *p *< 0.0001) at anterior sites than at posterior sites. Details of RF application CF values, LI drops, percentages of LI drop and RF delivery times, according to anatomical location, are reported in Figure 1.

### First-pass isolation and location of PV gaps

**Figure 2:**
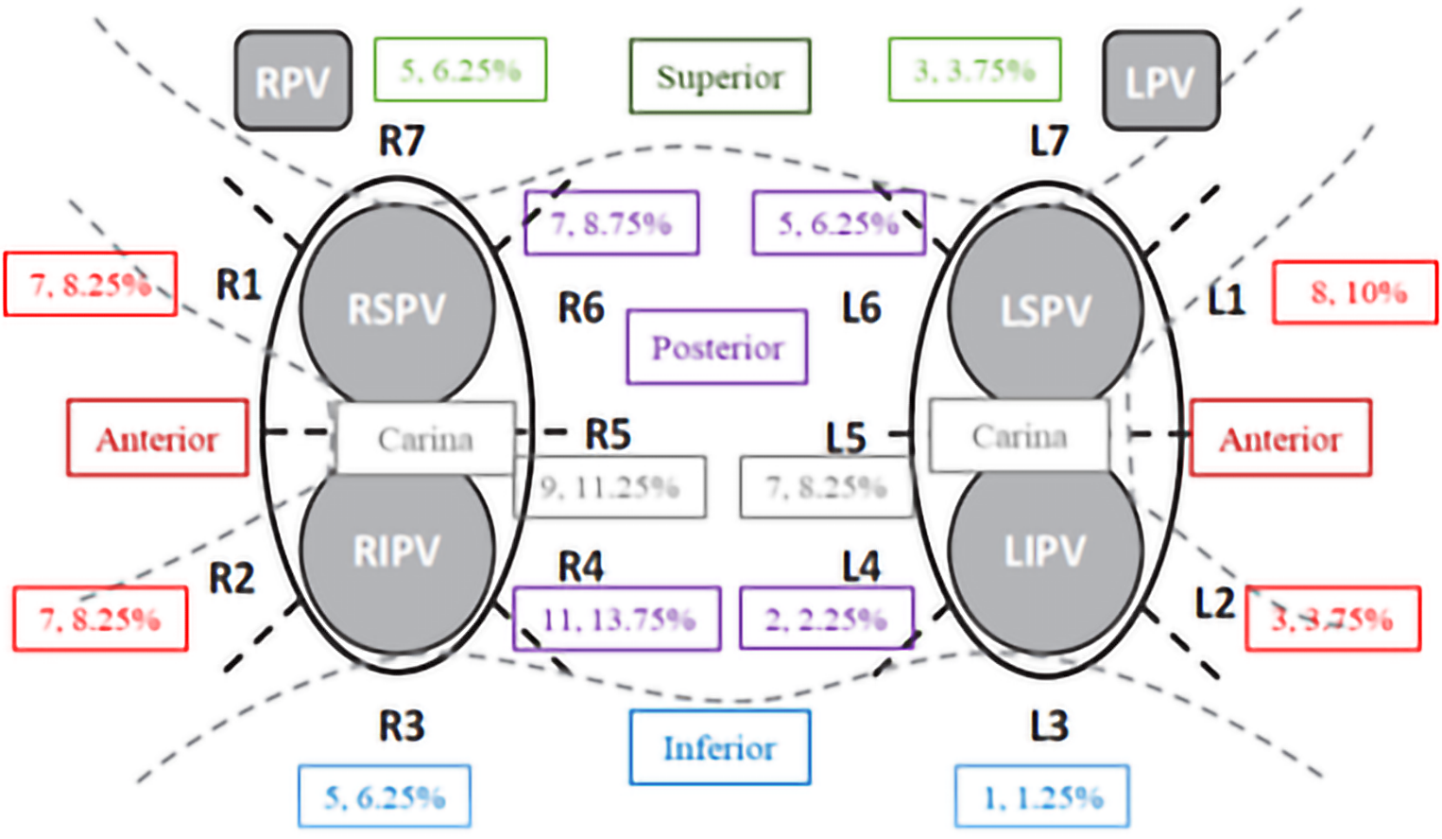
Distribution of PV gaps in patients without first-pass isolation.

**Figure 3:**
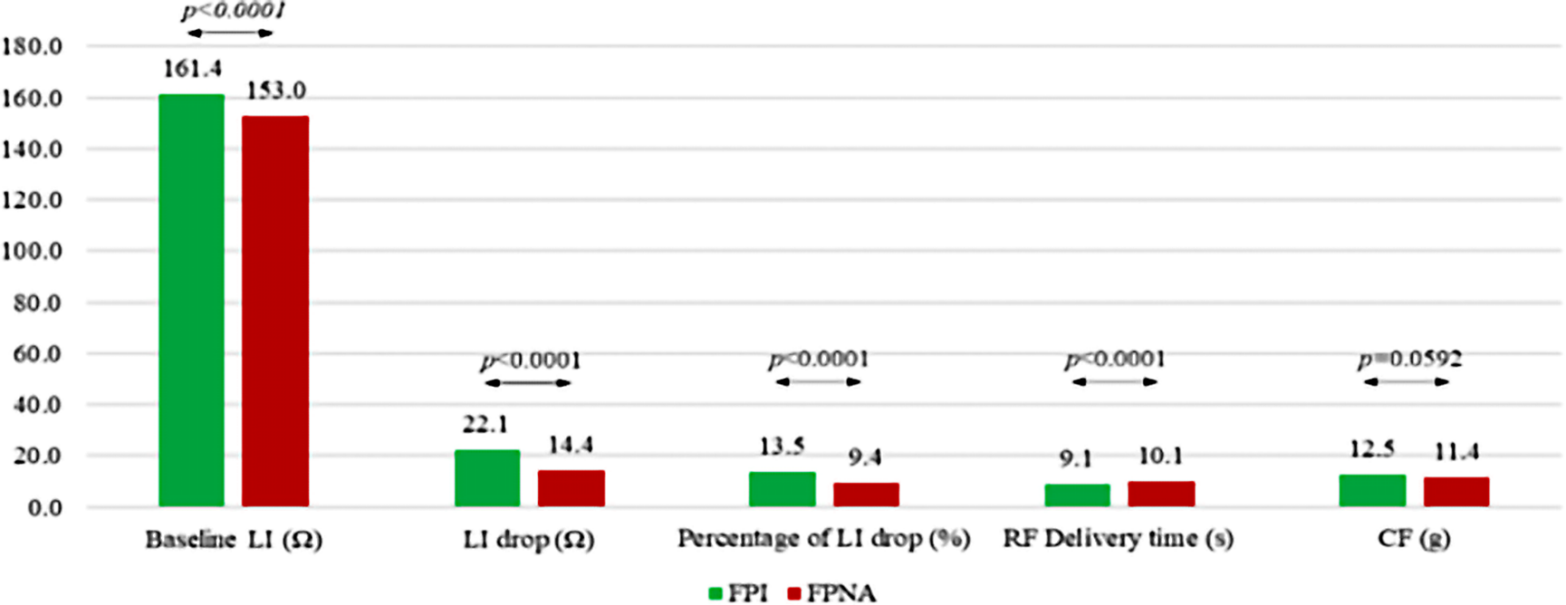
Procedural parameters at successful ablation spots and PV gaps (first-pass isolation sites vs. PV gap sites). FPI, first-pass isolation; FPNA, first-pass isolation not achieved.

The first-pass PV isolation rate per vein was 93.3% [in 180 out of 212 (84.9%) patients]. A total of 80 PV gaps were detected, mostly at the right PVs (63.7%, *p* = 0.013). PV gaps were located at anterior (*n* = 25, 31.3%), posterior (*n* = 25, 31.3%), carina (*n* = 16, 20%), superior (*n* = 8, 10%) and inferior (*n* = 6, 7.5%) sites, respectively. Details of the distribution of the PV gaps are depicted in Figure 2. At successful ablation spots, baseline LI, absolute LI drop and percentage of LI drop were greater than at PV gap spots (161.4 ± 19 Ω vs. 153.0 ± 13 Ω, *p *< 0.0001 for baseline LI; 22.1 ± 9 Ω vs. 14.4 ± 5 Ω, *p* < 0.0001 for LI drop and 13.5 ± 5% vs. 9.4 ± 3%, *p *< 0.0001 for percentage LI drop), whereas RF delivery time was shorter (9.1 ± 4 at successful ablation spots vs. 10.1 ± 4 s at PV gap spots, *p *< 0.0001). CF values were higher at successful ablation spots than at PV gap spots (12.5 ± 7 g vs. 11.4 ± 6 g, *p* = 0.0592) (Figure 3). On the basis of ROC analysis, the ideal LI drop, able to predict a successful ablation spot, was >20 Ω (Sensitivity = 56.8%, Specificity = 93.2%, PPV = 99.7%, Area under the ROC curve = 0.7841, *p *< 0.0001). Optimal LI drops were also identified for each left atrial anatomical region, being >21 Ω (60.2%, 93.5%, 99.8%, 0.8066, *p *< 0.0001) for anterior sites and >18 Ω (55.9%, 86.3%, 99.4%, 0.7368, *p *< 0.0001) for posterior sites. In terms of percentage LI drop, the ideal cut-off point was >12.5% (58.9%, 87.7%, 99.5%, 0.7782, *p *< 0.0001): > 14% (67.3%, 80.5%, 99.4%, 0.7949, *p *< 0.0001) for anterior sites and >12% (52.6%, 84.2%, 99.2%, 0.7315, *p *< 0.0001) for posterior sites. Logistic regression analysis was performed by setting successful lesions as the outcome. Every 5-point increment in LI drop was associated with successful ablation, with an OR of 2.03 (95% CI: 1.9–2.2, *p *< 0.0001; details are reported in Table 2). No steam pops or complications were reported during the procedures. At the end of the procedures, all PVs had been successfully isolated in all patients.

**Table 2:** Logistic regression analysis by setting successful lesions as the outcome.

Variable	Odds ratio	95% CI	*p*
LI drop (Ω)	1.152	1.1313–1.1731	<0.0001
Every 5-point increment in LI drop (Ω)	2.029	1.8534–2.2212	<0.0001
Percentage of LI drop (%)	1.2637	1.2266–1.3020	<0.0001

### Correlation between local impedance and key procedural parameters

**Figure 4:**
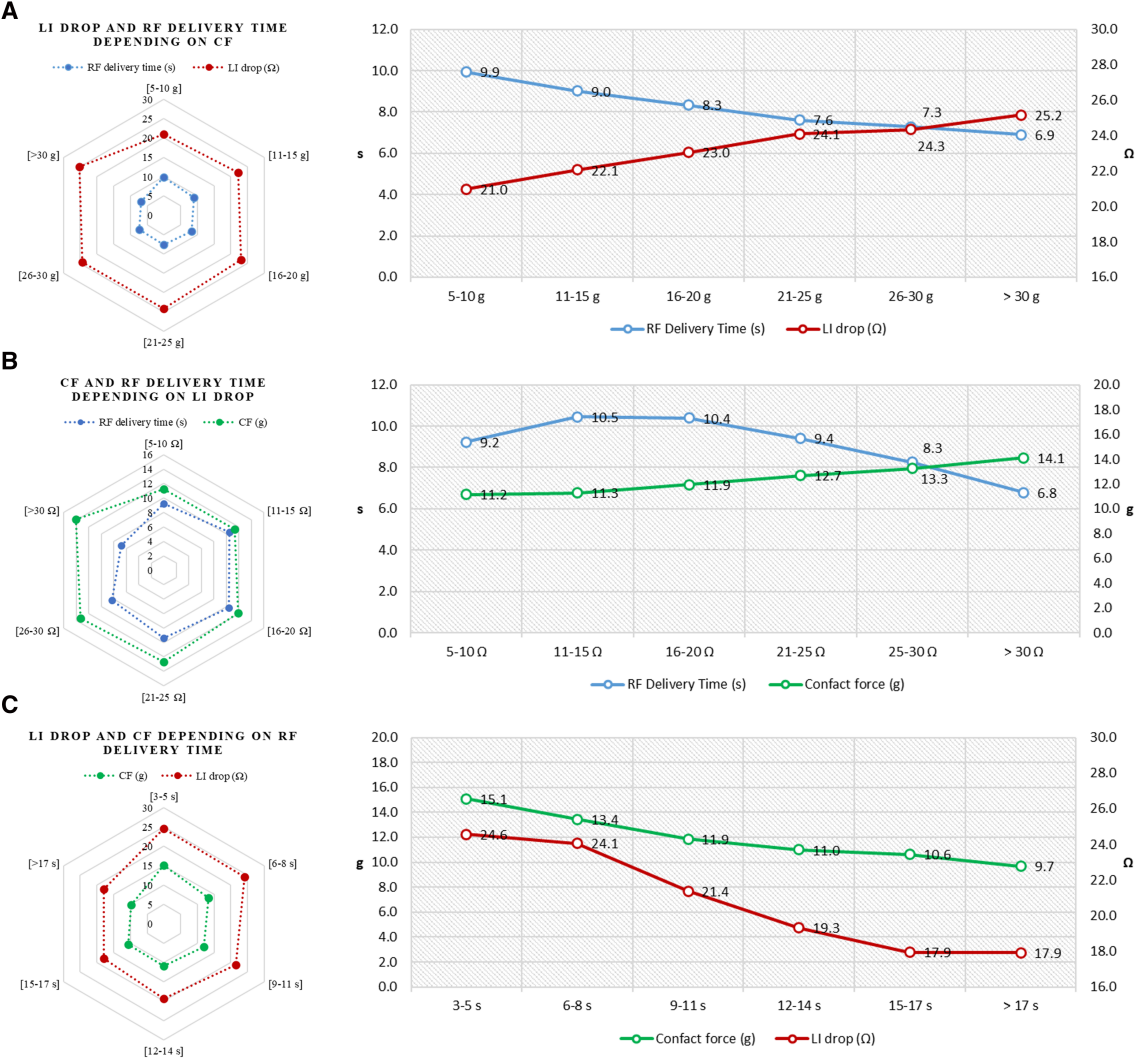
Correlation between key procedural parameters. Radar plots (on the left) showing the relationship between RF delivery time and CF values according to different degrees of LI drop (**A**); RF delivery time and LI drop values according to different degrees of CF (**B**); and LI drop and CF values according to different degrees of RF delivery time (**C**). Details of relationships among the three key parameters (on the right): RF delivery time and LI drop according to different levels of CF (**A**); RF delivery time and CF according to different degrees of LI drop (**B**); and CF and LI drop according to different values of RF delivery time (**C**).

The mean RF delivery time was 9.2 ± 4 s (median RF delivery time of 8.3 [6.0–11.8] s) and the mean CF was 12.5 ± 7 g (median CF of 10.5 [7.5–15.1] g). The higher the baseline LI was, the shorter the RF ablation time to the target drop was (*r* = −0.33, 95% CI: −0.34 to −0.31, *p *< 0.0001). Increasing CF showed a trend towards lower baseline LI (*r* = 0.02, 95% CI: 0.01–0.04, *p *= 0.0045). A non-linear association, with a weak correlation, emerged between the magnitude of LI drop and CF (*r* = 0.14, 95% CI: 0.13–0.16, *p *< 0.0001) whereas both CF and LI drop were inversely related to DT (*r* = −0.22, 95% CI: −0.23 to −0.20, *p *< 0.0001 for CF; *r* = −0.27, 95% CI: −0.29 to −0.26, *p *< 0.0001 for LI drop) (Figure 4). Details of the relationships among LI drop, CF and DT values, stratified by different intervals, are reported in Supplementary Figures S1, S2.

## Discussion

### Main findings

In this multicenter prospective study, PV isolation by means of an ablation catheter with integrated CF- and LI-sensing capabilities was achieved in all patients, causing no steam pops or major complications. An LI drop >21 Ω at anterior and >18 Ω at posterior anatomical sites predicted an acutely successful application. A higher CF was associated with an increased likelihood of optimal LI drop. Combining good CF with an adequate LI drop enabled RF delivery time to be reduced.

### The importance of a durable, transmural lesion

While several ablation approaches have been proposed, PV and/or PV antrum isolation remains the cornerstone of AF ablation. Although procedural success is achieved in almost all patients, the arrhythmia may recur, mainly owing to the lack of permanent, transmural electrical isolation of the PVs. Thus, the creation of high-quality lesions should improve the clinical outcome of AF ablation; for this reason, optimizing the available technology has been a constant challenge in catheter ablation.

### Ablation guided by local impedance

In previous studies, GI has proved to be related to RF-induced lesion dimension (3, 13), and an impedance fall ≥10 Ω is usually considered a marker of adequate lesion formation. LI measurement has the potential to increase the accuracy of lesion characterization and improve the outcome of RF ablations (14) by acting as a viable real-time indicator of tissue characteristics and predicting the durability of the lesions created (6–9). Previous studies (6–8) have demonstrated that: (1) LI is related to local tissue voltage and is higher in high-voltage areas; (2) the higher the basal LI value, the greater the LI drop during ablation; (3) the magnitude of the LI drop is proportional to the likelihood of achieving effective ablation; (4) an LI-guided ablation strategy presents a 100% rate of acute procedural effectiveness, without major complications, and, in a mixed population of paroxysmal and persistent AF patients, the AT/AF recurrence-free rate without a blanking period reaches >88% at 12 months. The additional value of LI may lie in the 4 times greater working range of LI, which allow a more precise titration of energy delivery (10) than generator impedance, especially during high power application when tissue heating occurs rapidly.

### Ablation guided by contact force and local impedance

The new Stablepoint™ ablation catheter can record LI together with CF, thereby allowing both mechanical contact and electrical coupling to be evaluated and enabling safe and effective lesions to be created (9). In our study, CF greater than 25 g between the catheter tip and the tissue did not have a major impact on LI drop. In addition, the overall data on the initial LI, LI drop and percentage LI drop were significantly higher in the success tags than in the failure tags. With the Stablepoint™ catheter, the optimal LI cut-off values seem to be higher than those obtained with other ablation catheters, such as the IntellaNav MiFi OI catheter (Boston Scientific, Marlborough, MA, USA). With this latter ablation catheter, Das et al. (7) reported that the optimal LI cut-off value was 16.1 Ω (PPV for block: 96.3%) for anterior/roof segments and 12.3 Ω (PPV for block: 98.1%) for posterior/inferior segments. In a pilot study involving 8 patients with paroxysmal or persistent AF, Szegedi et al. (15) demonstrated for the first time that measuring LI by means of the Stablepoint^TM^ catheter could predict optimal lesion formation. A local impedance drop >21.80 Ω on the anterior wall and >18.30 Ω on the posterior wall significantly increased the probability of creating a successful lesion. Similarly, in an LI-blind prospective study, Fukaya et al. (16) found that an insufficient LI drop, as measured by the Stablepoint™ catheter, was associated with gap formation during PVI, and that the best cutoff values for the LI drop and percentage LI drop were 20.0 Ω and 11.6%, respectively. In the present, large, multicenter study, our analysis of 13,891 ablation points confirmed these data and suggested new ideal LI drops of >21 Ω for the anterior and >18 Ω for the posterior left atrium. A non-linear association, with a weak correlation, emerged between the magnitude of LI drop and CF, therefore higher the CF at posterior sites did not translate into higher LI drop. LI is a direct measure of the resistive load at the catheter–tissue interface and, therefore, how the subsequent RF application is capable of creating significant resistive heating. It is well recognized that there are markedly different wall thicknesses in different regions of the LA, with thicker tissue present anteriorly, particularly at the left pulmonary vein/LA appendage ridge, and thinner tissue in the posterior region (7) and underlying tissue thickness could be a driver of the LI drop.

### Efficiency in radiofrequency AF ablation

The right combination between local impedance and contact force may make the AF ablation procedure more efficient. When dealing with a very well-established procedure such as PVI, and in competition with single-shot and established RF energy delivery techniques, procedural efficiency, such as first-pass PVI, is very important. The CF information facilitated better tissue contact for each RF lesion, and the Directsense™ local impedance information through the Directsense^TM^ technology allowed precise titration of RF to the underlying tissue with energy tailored to the evolving effects in real time. This ensures first-pass isolation of both vein pairs, thereby greatly reducing RF time. The proposed approach results in a very high rate of first-past isolation, despite the delivery of a small amount of RF. Taraji et al. (17) described an ablation protocol (CLOSE) that respected strict criteria for lesion depth and contiguity using predefined ablation index cut-offs of 400 at the posterior wall and 550 at the anterior wall. This strategy resulted in a high rate of acute PV isolation and a low recurrence rate. In addition, a recent large multicenter study that systematically employed the CLOSE protocol reported a first-pass PV isolation rate of 82.4% (18). The results of the present study are comparable in terms of acute PV isolation. However, the RF required in order to achieve these results was significantly lower. Indeed, the mean RF delivery time was 6 ± 2 and 5 ± 2 min (for right and left PV, respectively) compared with 16 ± 4 and 18 ± 6 min in the CLOSE study, and the mean total RF time was 11 ± 4 min, compared with 35.2 ± 11.1 in the VISTAX trial. Although caution is mandatory when comparing different technologies, the low values of RF needed to isolate the PVs probably stem from the efficiency of the proposed method, which uses a multiparametric approach to guide PV isolation. Moreover, the high power setting applied (≥ 45 W) increased procedural efficiency in comparison with our previous experience (6, 8) with an LI-guided approach through the IntellaNav MiFi OI catheter (35 W), in that the average RF delivery time was reduced to a third (9.2 ± 4 s vs. 31 ± 23 s). Although Ablation Index, Force-Time-Integral, and Lesion Size Index have contributed to improving the quality of PVI, a major limitation is that they do not consider tissue information. Beyond the ablation index-guided approach, which includes catheter-tip to tissue contact force, power and ablation duration, AF patients may benefit from further personalized ablation strategies. Apart from local impedance, adapting the ablation index to the left atrial wall thickness may also allow lower RF delivery, less fluoroscopy and shorter procedure times, while yielding a high rate of AF-free survival, as demonstrated by Teres et al. (19).

### Multiparametric approach to guide pulmonary vein isolation

In a national pilot study (9) we demonstrated that CF significantly impacts on effective lesion formation during RF PVI and the benefit of higher than 25 g contact between the catheter and the tissue appears to have less impact on LI drop (9). LI values that we used were empirically chosen and, due to the number (*n* = 45) of cases evaluated, we were unpowered to carry out advanced analyses, such as first pass isolation and regional ablation target. With our current large, international analysis of consecutive *de novo* AF cases, we demonstrated that an ablation strategy guided by LI and CF information results in a very high first pass isolation rate and LI drop is predictive of PV segment isolation. On the basis of the present findings and those of previous studies on LI technology (6–11), a multiparametric approach based on LI and CF measurements seems highly efficient in guiding effective PV isolation. Before starting RF delivery, we should search for adequate electromechanical coupling, aiming to obtain a minimum CF ≥ 5 g and a minimum baseline LI ≥ 100 Ω. RF energy is then applied in the power-controlled mode (45–50 W) with a temperature limit of 43°C, the aim being to achieve an LI drop of >21 Ω on the anterior and >18 Ω on the posterior wall within 10 s. RF delivery time seems to contribute more than CF in resulting LI drop thus, only in the event of failure to achieve the target LI drop, CF may be increased up to a value of 25 g.

### Limitations

This study had some limitations. Firstly, it was non-randomized. No deviation from the clinical practice of each center and operator was required. Although all patients underwent the same ablation protocol, some aspects, such as pre-procedural imaging and oral anticoagulant management, were not standardized. However, this observational prospective study may provide a representative image of the real-life scenario regarding the use of CF-LI technology in AF ablation. Secondly, esophageal temperature monitoring was not performed. However, no steam pops or major complications, including atrio-esophageal fistula or tamponade, occurred during or after the procedures. We cannot compare contact-force LI sensing with other technologies since different catheter types, measurements from different methods (e.g., generator impedance vs. local impedance) and proprietary software (e.g., DirectSense), may lead to different results. Lastly, we evaluated the impact of CF and LI on acute PV isolation. No data are as yet available on its impact on the medium- and long-term outcome.

## Conclusion

On the basis of the present multicenter prospective study of AF patients undergoing transcatheter ablation by means of a catheter with CF- and LI-measurement capability, an LI drop >21 Ω on the anterior and >18 Ω on the posterior left atrial wall predicts the achievement of first-pass PV isolation. A higher CF was associated with an increased likelihood of ideal LI drop. Combining good CF with an adequate LI drop allows RF delivery time to be significantly reduced.

## Data Availability

The data underlying this article will be shared on reasonable request to the corresponding author.
