# Supplementary material for: Insight into contact force local impedance technology for predicting effective pulmonary vein isolation
Source: Front Cardiovasc Med. 2023 Jul 5;10:1169037. doi: 10.3389/fcvm.2023.1169037 (PMC10354239; doi:10.3389/fcvm.2023.1169037)
Supplement: Supplementary file 1 [file Table1.docx]

**Supplementary Figure 1.** Relationships among contact force, local impedance and radiofrequency delivery time according to different intervals. Results from the correlation between the percentage LI drop and the other parameters are similar to those presented above (Percentage of LI drop: r = -0.204, 95%CI: -0.202 to -0.188, vs RF Delivery time, p < 0.0001; CF: r = 0.156, 95%CI: 0.139 to 0.172, vs CF, p < 0.0001). LI drop, CF and DT values were also stratified by different combinations (by 10 Ω increments in LI drop, 10 g increments in CF and 6-second increments in DT) to assess potential inferences. At different CF intervals (5-15 grams vs 16-25 grams vs > 25 grams), LI drops markedly increased from 21.4±8Ω to 23.4±9Ω and to 24.7±9Ω (all comparisons p < 0.0001) whereas RF DT significantly decreased (from 9.6±4 s to 8.1±4 s and to 7.1±3 s, all comparisons p < 0.0001) (Supplementary Figure 1, Panel A). On stratifying by LI drop interval, the greater the LI drop, the greater the CF was; by contrast, only when the LI drop was greater than 25 Ω did we observe a significant difference in terms of RF DT (Supplementary Figure 1, Panel B). A correlation between shorter RF DT and both greater LI drop and higher CF emerged: 24.2±10Ω for LI drop and 13.9±8 g for CF at 3-8 seconds of DT interval vs 20.6±7Ω and 11.5±6 g at 9-15 seconds of DT interval vs 17.9±6Ω and 10.3±5 g at >15 seconds of DT interval (all comparisons p<0.0001) (Supplementary Figure 1, Panel C). The greatest LI drops were achieved within 8 seconds.
