# Supplementary material for: Insight into contact force local impedance technology for predicting effective pulmonary vein isolation
Source: Front Cardiovasc Med. 2023 Jul 5;10:1169037. doi: 10.3389/fcvm.2023.1169037 (PMC10354239; doi:10.3389/fcvm.2023.1169037)
Supplement: Supplementary file 2 [file Table2.docx]

**Supplementary figure 2.** Multidimensional relationship between RF delivery time, CF values and LI drop values.
