# Supplementary figures and images for: Insight into contact force local impedance technology for predicting effective pulmonary vein isolation
Source: Front Cardiovasc Med. 2023 Jul 5;10:1169037. doi: 10.3389/fcvm.2023.1169037 (PMC10354239; doi:10.3389/fcvm.2023.1169037)

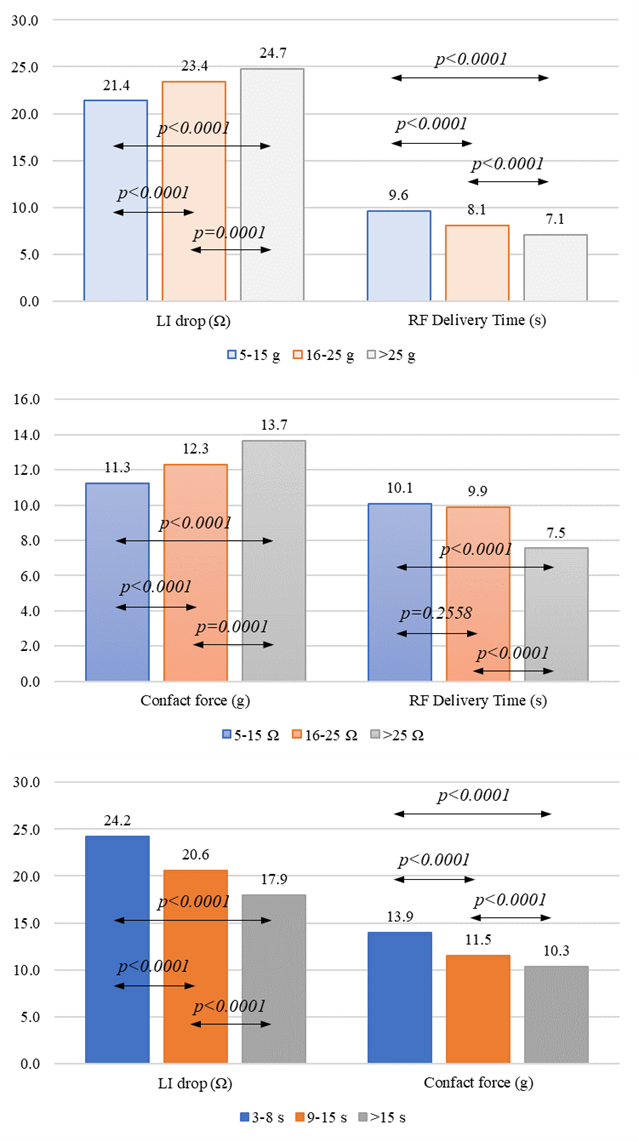

Supplement: Supplementary file 3 [file Image1.tif]

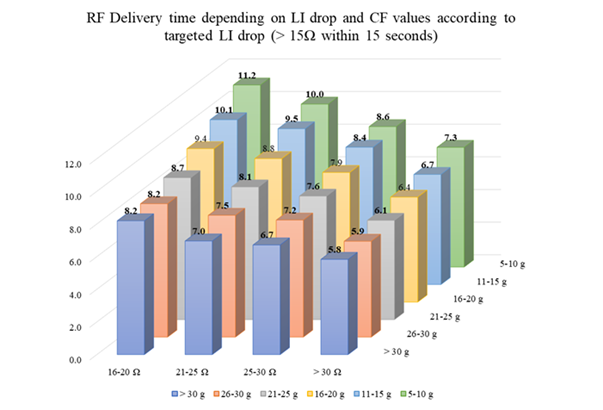

Supplement: Supplementary file 4 [file Image2.tif]
